# Is neck and shoulder posture, muscle activity and discomfort influenced by tablet inclination in young adults with and without neck pain?

**DOI:** 10.1371/journal.pone.0322207

**Published:** 2025-04-29

**Authors:** Petcharatana Bhuanantanondh, Siriyaphorn Rungkitlertsakul, Jim Richards

**Affiliations:** 1 Faculty of Physical Therapy, Mahidol University, Nakhon Pathom, Thailand; 2 Department of Physical Therapy, School of Integrative Medicine, Mae Fah Luang University, Chiang Rai, Thailand; 3 Allied Health Research unit, School of Health, Social Work and Sport, University of Central Lancashire, Preston, United Kingdom; Universidade de Aveiro Escola Superior de Saude de Aveiro, PORTUGAL

## Abstract

This study aimed to investigate the effect of tablet inclination on neck and shoulder posture, muscle activity, and discomfort in young adults with and without neck pain during a prolonged writing task. Participants performed a continuous writing task on a tablet for 40 minutes under two conditions, tablet lying flat and with a 30^o^ inclination. The results showed that young adults with neck pain demonstrated higher neck-shoulder muscle activity and discomfort whilst maintaining a similar neck-shoulder posture than those without neck pain. The 30^o^ inclination improved neck-shoulder posture and reduced neck discomfort but induced greater shoulder muscle activity. After 20 minutes, the flat tablet led to increased neck muscle activity in the neck pain group and increased neck discomfort in the group without neck pain. In conclusion, young adults should be recommended to use a 30^o^ inclination and writing on a flat tablet for longer than 20 minutes should be discouraged.

## Introduction

Neck and shoulder pain can contribute to disability in the long term [1] there are prevalent musculoskeletal disorders which have been linked to mobile device use, which include tablets [2–4]. During tablet use, individuals have been shown to adopt a more awkward posture than when using computers or laptops, which has been linked to increased likelihood of having neck-shoulder problems [5]. Across the life course, young adulthood may be considered as a critical period for developing or coping with musculoskeletal disorders [6].

Altered motor control in the cervical muscles has been reported with the presence of neck pain [7–9]; however, the specific changes in cervical muscle activation vary among individuals [8]. Individuals with neck pain typically have impaired neck proprioception causing changes in neck biomechanics and discomfort [9]. Moreover, individuals with neck pain showed different biomechanics and muscle activity compared with healthy individuals including greater neck flexion [10] and increases in Cervical Erector Spinae [CES] and Upper Trapezius [UT] muscle activity [11, 12].

Extended duration of use of mobile devices could lead to muscle fatigue [13] and posture adjustment [14] as well as increases in level of discomfort [15], with the use of mobile devices when seated for 30–45 minutes showing greater levels of discomfort [16]. It has also been reported that young adults who use mobile devices continuously for more than 30 minutes on a regular basis tend to develop musculoskeletal disorders [17].

Tablet inclination has been shown to raise the viewing angle and reduce neck flexion respectively; nevertheless, more shoulder flexion and shoulder discomfort have been reported [18–20]. Postural changes influenced by tablet inclination have been shown to affect neck and shoulder muscle activity [21, 22], but despite such findings being reported in the literature there is a lack of understanding of neck and shoulder biomechanics between young adults with and without neck pain during prolonged tablet writing and the association with discomfort.

To the best of the authors’ knowledge, differences in biomechanics, muscle activity and discomfort have not been explored between young adults with and without neck pain when using a tablet at different inclinations with prolonged writing. Such information would be useful to provide clearer evidence based ergonomic recommendations, in particular to those individuals that have neck pain. Therefore, this study aimed to determine neck and shoulder posture, muscle activity, and levels of discomfort between young adults with and without neck pain during a 40-minute writing task with the tablet lying flat and with a 30^o^ inclination. We hypothesized that there would be significant differences in the measures of neck and shoulder posture, muscle activity and discomfort between young adults with and without neck pain and significant changes with tablet inclination. Such information may help to give useful information to update and inform ergonomic recommendations.

## Methods

### Participants

This cross-sectional study aimed to compare neck and shoulder posture, muscle activity and discomfort between young adults with and without neck pain during tablet writing tasks at 0^o^ and 30^o^ inclinations across four 10-minute time intervals. G Power software was used to calculate the sample size with the level of confidence and power set as 0.05 and 80% respectively. The effect size was calculated based on Xie et al. [12] who reported a mean ± standard deviation of normalized UT muscle activity in young adults with neck-shoulder pain of 10.13 ± 7.95 and 5.14 ± 4.0 in those without neck-shoulder pain, which yielded a sample size required of 27 participants in each group. The inclusion criteria were aged between 18–25 years, right-handed dominant, having at least a year of experience of tablet use, normal or correctable vision with glasses, and currently using a tablet for at least 2 hours/day. The exclusion criteria were any prior injuries to the neck and/or upper extremities in the 12 months prior to the study, any systematic diseases, neurological problems, cardiovascular diseases, hypersensitivity to alcohol, or not able to communicate in Thai. The recruitment period for this study started from November 15, 2021 to June 30, 2022.

All participants who met the criteria completed two questionnaires: a modified version of the Nordic Musculoskeletal Questionnaire [23], and the Neck Disability Index (NDI) [24]. Participants were allocated to the neck pain group if they had neck pain relating to mobile device use that occurred during the 7-day period preceding the study; furthermore, they also had to report at least 8/100 score on the NDI [12], otherwise, they were allocated into the no neck pain group. Before enrolling in the study, all participants gave written informed consent. This study was approved by the Mahidol University Central Institutional Review Board (MU-CIRB 2021/204.2604).

### Procedures

A workstation was customized to fit with each individual’s anthropometry. The chair height was set so that their thighs were parallel to the ground and their feet were flat on the floor [25]; in addition, the table height was set to 5 cm above their resting-elbow level [26], and a tablet (iPad Pro 2020 with 2nd-generation Apple Pencil, Apple Inc., USA) was positioned 10 cm away from and parallel to the table edge [26].

To measure neck and shoulder flexion-extension, Inertial Measurement Units (IMU) sensors were attached to the middle of the forehead and on the middle of the upper arm on the right side respectively. To measure muscle amplitude, the Surface Electromyography (SEMG) sensors were applied according to the European recommendations for SEMG [27] with the sensor for CES positioned 2 cm lateral to the spinous process of the 4^th^ cervical vertebra, UT positioned at the midpoint between the acromion process and the spinous process of the 7^th^ cervical vertebra, and Anterior Deltoid (AD) positioned 2 cm away from the anterior edge of the muscle and 3 cm below the anterior rim of the acromion process. To measure discomfort, participants rated neck and shoulder pain on a Visual Analogue Scale (VAS), and a polar heart rate sensor was placed below the chest muscles to record Heart Rate Variability (HRV).

The baseline IMU and SEMG data were recorded before each writing condition with the participants sitting on the adjusted chair with a straight alignment of their neck and arms at their sides for a minute. For discomfort baseline, neck and shoulder VAS were rated before writing and HRV baseline was collected with the participant sitting comfortably on the chair using the backrest for 5 minutes.

**Fig 1:**
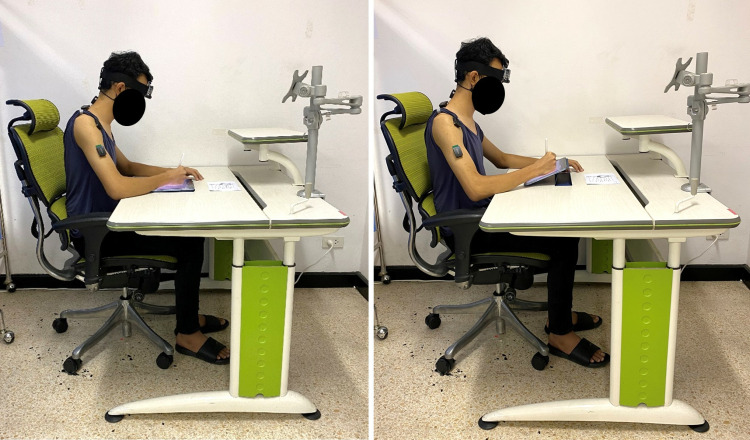
Writing on a tablet with 0° (left) and 30° (right) inclinations.

Participants performed continuous tablet writing tasks under both conditions (0^o^ and 30^o^ inclinations), Fig 1, for 40 minutes under each condition which has previously been used by Rungkitlertsakul et al. [28]. Before each writing condition, participants were asked to stand and stretch their bodies for 5 minutes to provide a washout period between conditions [29]. During each 10-minute interval, linear acceleration and muscle activity were recorded for a minute at the initial, middle, and end points. Average values from these three points were taken to represent the data for that interval. The VAS and HRV data were recorded at the end and the last 5 minutes of each interval respectively.

### Data analysis

Neck and shoulder flexion/extension were calculated from acceleration respecting to X, Y, and Z axes (a_x_, a_y_, and a_z_) which were filtered using a 0.2 second moving average. The formula for neck flexion/extension was “angle= tan^-1^(a_z_/ a_y_)” while that for shoulder flexion/extension was “angle = tan^-1^(a_x_/ a_y_)”. Positive and negative values denoted flexion and extension respectively.

Raw EMG signals, with a 1200Hz sampling frequency and a 20–450 Hz bandpass filter, were processed by correcting for the DC offset, rectifying, and low pass filtering with a 2^nd^ order Butterworth filter with a 20 Hz cutoff frequency using the EMGworks® Analysis Software (Delsys Inc., USA). The average data under each condition was normalized to the maximum observed signal for each muscle in all conditions over the four time points.

For HRV data, the medium artefact correction with 5% acceptance threshold and 500-lamba smoothness priors by the Kubios HRV Standard software (Kubios Oy, Finland) were performed. Then, the spectrum estimation was applied to find the Ratio of low frequency and high frequency (LF/HF). High and low LF/HF indicated high and low discomfort respectively.

### Statistical analysis

All statistical analyses were performed using SPSS version 22 (IBM, USA). The Shapiro-Wilk test found that the data were non-normally distributed. Accordingly, Mann Whitney U, Wilcoxon signed-rank and Friedman tests were used to investigate differences between groups, tablet inclinations and time intervals respectively, and the median and interquartile range (IQR) were used for descriptive statistics. If a significant difference between time intervals was found, pairwise comparisons were conducted using Wilcoxon Signed Rank tests. The significant level was set at α = 0.05.

## Results

Fifty-four right-hand dominant participants were recruited. No significant differences between groups were seen in the demographic data with the exception of the NDI score, Table 1. Data for neck and shoulder posture, muscle activity, and discomfort at baseline between groups and tablet inclinations are presented in Table 2. (S1 File) Mann-Whitney U tests revealed significant differences between groups at baseline of both neck VAS at 0^o^ inclination (P < 0.01) and 30^o^ inclination (P = 0.001), non-dominant shoulder VAS at 0^o^ inclination and 30^o^ inclination (P = 0.020), and HRV at 0^o^ inclination (P = 0.008).

**Table 1:** Demographic data.

	Neck pain (n = 27)	No neck pain (n = 27)	P-value
	Median (IQR)		
**Age (years)**	20.00 (1.00)	20.00 (2.00)	0.274
**Weight (kg.)**	50.00 (12.00)	54.00 (10.00)	0.341
**Height (cm.)**	161.00 (8.00)	161.00 (8.00)	0.298
**BMI (kg./m2)**	19.51 (3.29)	20.45 (2.45)	0.139
**Neck Disability Index (points)**	14.00 (8.00)	0.00	<0.001*
**Tablet usage experience (years)**	2.00 (1.50)	3.00 (2.00)	0.136
**Regular tablet writing (hours/day)**	3.00 (4.00)	3.50 (4.00)	0.938
**Regular tablet writing (minutes/session)**	30.00 (40.00)	60.00 (37.50)	0.214
	**N**		
**Male: Female**	8:19	1:26	
**Regular exercise**			
**- Never**	3	2	
**- 1–3 times/month**	15	12	
**- 1–3 times/week**	9	11	
**- More than 1–3 times/week**	0	2	
**A tablet inclination used regularly during the writing**			
**- 0** ^**o**^	6	9	
**- 20**^**o**^ **-35**^**o**^	17	17	
**- 36**^**o**^ **-50**^**o**^	3	0	
**- 51**^**o**^ **-65**^**o**^	1	1	
**A tablet screen position during writing**			
**- Parallel to the edge of a table**	15	11	
**- Rotated to a writing hand**	12	16	

IQR = Interquartile range and * P <0.05 (Significant difference for Mann Whitney U test between groups)

**Table 2:** Baseline Comparisons in neck and shoulder posture, muscle activity, and discomfort between groups and tablet inclinations.

	Groups	Median (IQR)		P-value between tablet inclinations
		Tablet inclinations		
		0^o^	30^o^	
**Neck F (+)/ E (-) (** ^**o**^ **)**	**Neck pain (n = 27)**	–10.260 (8.720)	–10.860 (8.973)	0.341
	**No neck pain (n = 27)**	–5.685 (7.373)	–6.150 (5.930)	0.078
**P-value between groups**		0.109	0.072	
**Shoulder F (+)/ E (-) (** ^**o**^ **)**	**Neck pain (n = 27)**	–1.300 (6.260)	–0.015 (5.563)	0.568
	**No neck pain (n = 27)**	–0.475 (6.150)	–1.060 (5.860)	0.471
**P-value between groups**		0.511	0.993	
**Non-dominant (Lt.)** **Av. CES amplitude (Normalized)**	**Neck pain (n = 27)**	0.080 (0.070)	0.080 (0.053)	0.416
	**No neck pain (n = 27)**	0.080 (0.040)	0.080 (0.040)	0.475
**P-value between groups**		0.664	0.586	
**Dominant (Rt.)** **Av. CES amplitude (Normalized)**	**Neck pain (n = 27)**	0.070 (0.040)	0.060 (0.033)	0.757
	**No neck pain (n = 27)**	0.060 (0.050)	0.060 (0.040)	0.678
**P-value between groups**		0.242	0.424	
**Non-dominant (Lt.)** **Av. UT amplitude (Normalized)**	**Neck pain (n = 27)**	0.030 (0.030)	0.040 (0.033)	0.143
	**No neck pain (n = 27)**	0.010 (0.040)	0.010 (0.030)	0.884
**P-value between groups**		0.156	0.139	
**Dominant (Rt.)** **Av. UT amplitude (Normalized)**	**Neck pain (n = 27)**	0.020 (0.020)	0.020 (0.020)	0.130
	**No neck pain (n = 27)**	0.020 (0.010)	0.020 (0.010)	0.235
**P-value between groups**		0.346	0.060	
**Dominant (Rt.)** **Av. AD amplitude (Normalized)**	**Neck pain (n = 27)**	0.020 (0.040)	0.030 (0.023)	0.167
	**No neck pain (n = 27)**	0.020 (0.020)	0.020 (0.030)	0.584
**P-value between groups**		0.669	0.129	
**Non-dominant (Lt.)** **neck VAS**	**Neck pain (n = 27)**	0.000 (1.215)	0.000 (0.620)	0.059
	**No neck pain (n = 27)**	0.000	0.000	1.000
**P-value between groups**		<0.001*	0.001*	
**Dominant (Rt.)** **neck VAS**	**Neck pain (n = 27)**	0.000 (0.688)	0.000 (1.170)	0.515
	**No neck pain (n = 27)**	0.000	0.000	1.000
**P-value between groups**		0.002*	0.001*	
**Non-dominant (Lt.)** **shoulder VAS**	**Neck pain (n = 27)**	0.000 (0.000)	0.000 (0.000)	0.917
	**No neck pain (n = 27)**	0.000	0.000	1.000
**P-value between groups**		0.020*	0.020*	
**Dominant (Rt.)** **shoulder VAS**	**Neck pain (n = 27)**	0.000 (0.000)	0.000 (0.000)	0.715
	**No neck pain (n = 27)**	0.000	0.000	1.000
**P-value between groups**		0.078	0.078	
**HRV (LF/HF)**	**Neck pain (n = 27)**	1.440 (1.815)	1.390 (2.085)	0.530
	**No neck pain (n = 27)**	0.650 (0.480)	0.855 (1.135)	0.062
**P-value between groups**		0.008*	0.094	

IQR = Interquartile range, Av. = Average, F/E = Flexion/Extension, EMG = Electromyography, CES = Cervical Erector Spinae, UT = Upper Trapezius, AD = Anterior Deltoid, VAS = Visual Analogue Scale, HRV = Heart Rate Variability, LF/HF = Ratio of low frequency and high frequency, and * P < 0.05 (Significant difference for Mann Whitney U test between groups and Wilcoxon signed-rank test between tablet inclination

Neck and shoulder posture, muscle activity, and discomfort during the tablet writing were tested between groups using the Mann-Whitney U tests and between tablet inclinations using Wilcoxon Signed Rank tests, Table 3. Between groups, the neck pain group had notably greater amplitudes of both CES at 0^o^ (P < 0.001) and 30^o^ (P < 0.01), both UT at 0^o^ (P < 0.01) and 30^o^ (P < 0.05) and dominant AD at 0^o^ and 30^o^ (P < 0.001). In addition, greater discomfort was seen in the neck pain group for both neck and shoulder VAS at 0^o^ and 30^o^ (P < 0.001) and HRV at 0^o^ and 30^o^ (P < 0.05) compared to the no neck pain group. However, neck and shoulder posture were not significantly different between groups in either the 0^o^ or 30^o^ tablet inclinations. When compared to the 0^o^ tablet inclination, the 30^o^ inclination influenced both neck pain and no neck pain groups similarly with significant decreases in neck flexion (Neck pain: P = 0.019, No neck pain: P < 0.001), shoulder extension (Neck pain and No neck pain: P < 0.001) and both neck VAS (Neck pain: P < 0.01, No neck pain: P < 0.001) but with significantly greater dominant UT amplitude (Neck pain and No neck pain: P < 0.001). In the neck pain group, the 30° tablet inclination significantly increased dominant AD amplitude (P = 0.010) and decreased non-dominant CES amplitude (P = 0.010) compared to the 0° inclination. Although the medians of non-dominant UT amplitude and dominant shoulder VAS were similar between inclinations, the interquartile range (IQR) for non-dominant UT amplitude was significantly greater at the 30° inclination (P = 0.033), indicating increased variability. In contrast, the IQR for dominant shoulder VAS was significantly smaller (P = 0.005), reflecting reduced variability.

**Table 3:** Comparisons of average neck and shoulder posture, muscle activity and discomfort between groups and tablet inclinations.

	Groups	Median (IQR)		P-value between tablet inclinations
		Tablet inclinations		
		0^o^	30^o^	
**Neck F (+)/ E (-) (** ^**o**^ **)**	**Neck pain (n = 27)**	31.34 (18.90)	29.54 (18.91)	0.019*
	**No neck pain (n = 27)**	34.58 (11.34)	31.39 (11.08)	<0.001*
**P-value between groups**		0.066	0.571	
**Shoulder F (+)/ E (-) (** ^**o**^ **)**	**Neck pain (n = 27)**	–13.86 (18.83)	–8.18 (22.51)	<0.001*
	**No neck pain (n = 27)**	–13.39 (15.31)	–8.50 (8.43)	<0.001*
**P-value between groups**		0.191	0.769	
**Non-dominant (Lt.)** **Av. CES amplitude (Normalized)**	**Neck pain (n = 27)**	0.231 (0.074)	0.224 (0.078)	0.010*
	**No neck pain (n = 27)**	0.184 (0.135)	0.197 (0.130)	0.663
**P-value between groups**		<0.001*	0.008*	
**Dominant (Rt.)** **Av. CES amplitude (Normalized)**	**Neck pain (n = 27)**	0.200 (0.106)	0.201 (0.099)	0.396
	**No neck pain (n = 27)**	0.148 (0.079)	0.153 (0.091)	0.147
**P-value between groups**		<0.001*	<0.001*	
**Non-dominant (Lt.)** **Av. UT amplitude (Normalized)**	**Neck pain (n = 27)**	0.051 (0.049)	0.051 (0.065)	0.033*
	**No neck pain (n = 27)**	0.044 (0.045)	0.046 (0.041)	0.067
**P-value between groups**		0.003*	0.020*	
**Dominant (Rt.)** **Av. UT amplitude (Normalized)**	**Neck pain (n = 27)**	0.083 (0.054)	0.093 (0.065)	<0.001*
	**No neck pain (n = 27)**	0.063 (0.036)	0.073 (0.039)	<0.001*
**P-value between groups**		<0.001*	<0.001*	
**Dominant (Rt.)** **Av. AD amplitude (Normalized)**	**Neck pain (n = 27)**	0.043 (0.039)	0.049 (0.036)	0.010*
	**No neck pain (n = 27)**	0.035 (0.022)	0.035 (0.025)	0.837
**P-value between groups**		<0.001*	<0.001*	
**Non-dominant (Lt.)** **neck VAS**	**Neck pain (n = 27)**	1.88 (3.07)	1.23 (2.49)	0.003*
	**No neck pain (n = 27)**	0.00 (1.84)	0.00 (0.79)	<0.001*
**P-value between groups**		<0.001*	<0.001*	
**Dominant (Rt.)** **neck VAS**	**Neck pain (n = 27)**	1.93 (3.35)	0.76 (2.77)	<0.001*
	**No neck pain (n = 27)**	0.00 (1.73)	0.00 (0.65)	<0.001*
**P-value between groups**		<0.001*	<0.001*	
**Non-dominant (Lt.)** **shoulder VAS**	**Neck pain (n = 27)**	0.00 (2.06)	0.00 (1.45)	0.053
	**No neck pain (n = 27)**	0.00 (0.00)	0.00 (0.00)	0.367
**P-value between groups**		<0.001*	<0.001*	
**Dominant (Rt.)** **shoulder VAS**	**Neck pain (n = 27)**	0.00 (1.81)	0.00 (0.90)	0.005*
	**No neck pain (n = 27)**	0.00 (0.00)	0.00 (0.00)	0.943
**P-value between groups**		<0.001*	<0.001*	
**HRV (LF/HF)**	**Neck pain (n = 27)**	1.45 (1.82)	1.54 (1.82)	0.355
	**No neck pain (n = 27)**	1.16 (1.02)	1.26 (1.11)	0.187
**P-value between groups**		0.014*	0.039*	

IQR = Interquartile range, Av. = Average, F/E = Flexion/Extension, EMG = Electromyography, CES = Cervical Erector Spinae, UT = Upper Trapezius, AD = Anterior Deltoid, VAS = Visual Analogue Scale, HRV = Heart Rate Variability, LF/HF = Ratio of low frequency and high frequency, and * P < 0.05 (Significant difference for Mann Whitney U test between groups and Wilcoxon signed-rank test between tablet inclination

Table 4 shows the data over 40 minutes of tablet writing. In the neck pain group, the Friedman tests showed a significant main effect at the 0^o^ tablet inclination for neck flexion (P < 0.001), both CES (P < 0.01), dominant UT (P = 0.018), and both neck VAS (P < 0.01). Post Hoc Wilcoxon signed rank test showed neck flexion being significantly decreased from the 1^st^, 2^nd^, and 3^rd^ to 4^th^ intervals (P < 0.001). There were significant decreases from the 1^st^ to 4^th^ interval in non-dominant CES (P = 0.009), dominant UT (P = 0.016), and both neck VAS (P < 0.01). Dominant CES significantly increased from the 1^st^ to 3^rd^ (P = 0.011) and 2^nd^ to 3^rd^ intervals (P = 0.008). A significant main effect at the 30^o^ tablet inclination was seen in the non-dominant CES (P = 0.001) and dominant neck VAS (P = 0.003). Pairwise comparisons with adjusted p-values showed, non-dominant CES significantly increased from the 1^st^ to 3^rd^ (P = 0.013), 1^st^ to 4^th^ (P = 0.004), and 2^nd^ to 4^th^ (P = 0.037) intervals whereas dominant neck VAS significantly increased from the 1^st^ to 3^rd^ intervals (P = 0.037). In the group without neck pain, significant main effects at 0^o^ tablet inclination were seen in the dominant AD (P = 0.006) and both neck VAS (P < 0.001). Post Hoc Wilcoxon signed rank test showed significant increases in dominant AD from the 2^nd^ to 3^rd^ (P = 0.022) and 2^nd^ to 4^th^ (P = 0.043) intervals. Non-dominant neck VAS significantly increased from the 1^st^ to 3^rd^ (P = 0.043) and 1^st^ to 4^th^ (P = 0.037) while dominant neck VAS significantly increased from the 1^st^ to 4^th^ interval (P = 0.011). At the 30^o^ tablet inclination, there was a significant main effect only in HRV (P = 0.017) with the pairwise comparison with adjusted p-values demonstrating a significant increase from the 1^st^ to 4^th^ interval (P = 0.027).

**Table 4:** Significant changes over 40 minutes in neck and shoulder posture, muscle activity, and discomfort between groups and tablet inclinations.

Group	Tablet inclinations	Outcome	Median (IQR)				P-value (Friedman’s test)	Adjusted P-value (significant Pairwise comparisons)
			1^st^ interval	2^nd^ interval	3^rd^ interval	4^th^ interval		
**Neck pain** **(n = 27)**	**0**	**Neck F (+)/E (-) (** ^**o**^ **)**	32.78 (20.25)	35.75 (20.67)	34.05 (19.51)	24.95 (14.70)	<0.001*	1^st^ - 4^th ^< 0.001* 2^nd^- 4^th^ < 0.001* 3^rd^ - 4^th^ < 0.001*
		**Non-dominant (Lt.)** **Av. CES amplitude (Normalized)**	0.214 (0.071)	0.231 (0.074)	0.232 (0.076)	0.232 (0.087)	0.005*	1^st^ - 4^th^ = 0.009*
		**Dominant (Rt.)** **Av. CES amplitude (Normalized)**	0.194 (0.091)	0.193 (0.106)	0.208 (0.111)	0.202 (0.116)	0.001*	1^st^ - 3^rd^ = 0.011* 2^nd^ - 3^rd^ = 0.008*
		**Dominant (Rt.)** **Av. UT amplitude (Normalized)**	0.080 (0.043)	0.078 (0.052)	0.087 (0.054)	0.087 (0.052)	0.018*	1^st^ - 4^th^ = 0.016*
		**Non-dominant (Lt.) Neck VAS**	0.960 (2.230)	1.830 (2.860)	2.380 (2.860)	2.520 (2.990)	<0.001*	1^st^ - 4^th^ = 0.002*
		**Dominant (Rt.) Neck VAS**	1.090 (2.740)	1.830 (3.600)	2.010 (3.800)	2.360 (3.180)	0.002*	1^st^ - 4^th^ = 0.005*
	**30**	**Non-dominant (Lt.)** **Av. CES amplitude (Normalized)**	0.216 (0.074)	0.223 (0.088)	0.227 (0.091)	0.229 (0.075)	0.001*	1^st^ - 3^rd^ = 0.013* 1^st^ - 4^th^ = 0.004* 2^nd^ - 4^th^ = 0.037*
		**Dominant (Rt.) Neck VAS**	0.000 (1.760)	0.840 (2.580)	1.220 (3.370)	1.360 (3.470)	0.003*	1^st^ - 3^rd^ = 0.037*
**No neck pain** **(n = 27)**	**0**	**Dominant (Rt.)** **Av. AD amplitude (Normalized)**	0.033 (0.024)	0.038 (0.024)	0.035 (0.026)	0.033 (0.028)	0.006*	2^nd^ - 3^rd^ = 0.022* 2^nd^ - 4^th^ = 0.043*
		**Non-dominant (Lt.) Neck VAS**	0.000 (1.410)	0.000 (1.190)	0.920 (2.020)	0.540 (2.330)	<0.001*	1^st^ - 3^rd^ = 0.043* 1^st^ - 4^th^ = 0.037*
		**Dominant (Rt.) neck VAS**	0.000 (1.200)	0.000 (1.620)	0.000 (1.850)	0.420 (2.190)	<0.001*	1^st^ - 4^th^ = 0.011*
	**30**	**HRV (LF/HF)**	0.984 (1.155)	1.116 (1.132)	1.172 (1.458)	1.483 (1.222)	0.017*	1^st^ - 4^th^ = 0.027*

IQR = Interquartile range, Av. = Average, F/E = Flexion/Extension, EMG = Electromyography, CES = Cervical Erector Spinae, UT = Upper Trapezius, AD = Anterior Deltoid, VAS = Visual Analogue Scale, HRV = Heart Rate Variability, LF/HF = Ratio of low frequency and high frequency, * P < 0.05 (Significant difference for Friedman’s test), and Adjusted P-value for pairwise comparisons (Bonferroni correction)

## Discussion

According to baseline comparisons, young adults with neck pain had relatively more discomfort, including both neck VAS, non-dominant shoulder VAS and HRV, than those without neck pain. During the tablet writing, the neck pain group demonstrated greater neck-shoulder muscle activity in both CES, both UT, and dominant AD, and discomfort including neck-shoulder VAS and HRV than the group without neck pain. However, neck-shoulder posture did not significantly differ between groups. Increased neck-shoulder muscle activity in the neck pain group was consistent with previous studies [11,12,30]. Xie et al. reported that young adults with neck-shoulder pain had higher levels of CES and UT muscle activity than those without neck-shoulder pain during texting on a smartphone [12]; similarly, Leonard et al. found comparatively more UT amplitude during writing in young adults with neck pain than those without neck pain [11]. Altered motor control is a potential explanation for the increased muscle activity observed in individuals with neck pain. When muscles are injured and painful, the central nervous system may change muscle recruitment to reduce the use of the painful muscle but still exhibits a similar motor output [8,9,31]. Additionally, in individuals with neck pain, deep cervical muscle function is typically impaired; therefore, there was increased activation of superficial layers of muscles to maintain cervical stability [32]. Similarly to neck-shoulder VAS, LF/HF was higher in the neck pain group as compared to the group without neck pain. Hence, LF/HF could possibly be used to differentiate discomfort between those with and without neck pain. This is also supported by a previous systematic review which reported that HRV can be helpful to evaluate pain [33].

Although neck and shoulder posture were not significantly different between groups, the group with neck pain had slightly less neck flexion than the group without neck pain. However, this finding is in contrast to previous studies in terms of neck posture [10,34]. When compared to individuals without neck pain, Szeto et al. and Kim reported relatively more neck flexion during 10–15 minutes of computer work [34] and during 5 minutes of smart phone use [10] respectively. A possible reason for this inconsistency was the different usage duration. Duration in the current study lasted 40 minutes whereas Szeto et al. and Kim recorded neck posture for no more than 15 minutes. With extended duration, participants with neck pain might have difficulties enduring load and pain over such a long duration; therefore, they might adjust their neck to be in a more neutral position to alleviate excessive stress on the neck. Consequently, instead of increased neck flexion as compared to the group without neck pain, the neck pain group had less neck flexion in this study. According to previous studies, shoulder flexion and extension between young adults with and without neck pain during tablet writing were not compared. Accordingly, this would show that young adults with and without neck pain had a similar shoulder posture during writing. Overall comparisons between groups demonstrated similar postures but with greater CES, UT and dominant AD amplitudes. This possibly implied that young adults with neck pain generated more neck and shoulder muscle activity to maintain a similar neck-shoulder posture than those without neck pain. Increased neck and shoulder VAS in the neck pain group were also associated with more LF/HF as compared to the group without neck pain.

Both groups exhibited less neck flexion, shoulder extension, and neck VAS when using the tablet at a 30° inclination compared to the flat tablet. However, dominant UT muscle activity was higher at the 30° inclined tablet than at the flat tablet in both groups. Decreased neck flexion with increased inclination in this finding supported previous studies [18–20]. Despite decreased neck flexion by inclining a tablet to 30^o^, both groups still exhibited greater neck flexion, 20^o^ [35]. However, shoulder extension decreased with the inclined tablet whereas Young et al. found increased shoulder flexion with increased tablet inclinations [22]. This contrast in findings might result from the restriction of using a backrest in the current study which was permitted in the study by Young et al. Due to restriction of using a backrest, participants tended to lean forward which would reduce the distance between their body and the tablet. Hence, participants in this study extended their shoulders rather than flexing. Decreases in neck VAS and increases in dominant UT muscle activity when inclining the tablet in both groups did not support the study of Chui et al. They reported no change of neck-shoulder VAS among various tablet inclinations [21]. The different findings were possibly caused by insufficient duration in the previous study (15 minutes) to induce discomfort. Chui et al. also found UT muscle activity decreased with increased tablet inclinations [21]. This contrast in findings might be due to differences in table height which could vary the screen height between studies. UT activation could increase due to either low or high screen height. Because of a higher working surface, individuals possibly elevated their shoulder which would require greater UT activation [36]. Also, more UT muscle activity was possibly induced by a low screen height because it assisted holding the head during prolonged deep neck flexion [37]. Our findings also revealed that a tablet with 30^o^ inclination reduced non-dominant CES muscle activity and dominant shoulder VAS but induced greater non-dominant UT and dominant AD muscle activity. Therefore, it could be implied that a tablet with a 30^o^ inclination is beneficial to reduce biomechanical load on the neck leading to less discomfort. Nevertheless, it induced greater UT and AD muscle activity particularly in the neck pain group. Moreover, LF/HF did not differ between tablet inclinations in both groups, which did not correspond to previous studies [38, 39]. Le and Marras [38] reported significantly higher LF/HF during standing compared to sitting, whereas our study involved only seated conditions. Weston et al. [39] discovered that the chair (reclined and regular chairs) and the device (computer and tablet use) had a significant impact on LF/HF, with the least LF/HF shown in the reclined chair during tablet use. However, in our study, both conditions used the same workstation setup, including chair and table. A sitting position and a fixed workstation may result in slight differences in posture across conditions in our study, which would not create enough differences in physiological discomfort to alter HRV between tablet inclinations.

When considering changes over 40 minutes for the neck pain group with the 0^o^ tablet inclination, the dominant CES increased between 20–30 minutes. This was followed by increases in non- dominant CES, dominant UT, and both neck VAS with a reduction in neck flexion from 30 to 40 minutes. At the 30^o^ tablet inclination, the neck pain group also showed increases in non-dominant CES and dominant neck VAS after 20 minutes. In terms of CES and neck flexion, our findings did not correspond with Szeto et al., as we found changes in CES muscle activity and neck flexion only in the neck pain group, with Szeto et al. reporting that young adults without neck pain showed decreases in neck flexion but increased CES over 30 minutes of tablet use [14]. This was possibly due to different usage configurations. In the study of Szeto et al., participants were instructed to hold a tablet with both hands whereas participants in the current study placed a tablet on the table. Although neck VAS at the 0^o^ tablet inclination in both groups tended to increase after 20 or 30 minutes, HRV showed a significant increase from 10 to 40 minutes only in the group without neck pain. This was consistent with the study of Le and Marras [38], who reported a minimally increasing trend of the LF/HF while sitting and typing on a computer for an hour. Therefore, HRV can be a sensitive measure for detecting changes in discomfort over extended duration in young adults without neck pain.

This study offered a comprehensive investigation in terms of both biomechanics and physiological variables and controlled confounding factors such as the tablet size, task instruction and temperature. However, there were still some limitations. Although this study considered the effect of tablet writing on the neck and shoulder, it did not consider other spinal regions such as the thoracic and lumbar regions which could influence cervical biomechanics [40]. The majority of neck pain participants recruited in this study only had mild neck disability (NDI = 5–14 points). The inclusion of young adults with moderate to severe neck disability should also be considered in future studies as different levels of neck disability may yield different findings. Future studies should consider the biomechanics of the whole spine to determine if the tablet inclination contributes a benefit or drawback to other spinal regions.

## Conclusion

The findings of this study would suggest that, when compared to a 0^o^ tablet inclination, a 30^o^ inclination should be recommended to improve neck-shoulder posture and discomfort for young adults with and without neck pain; although, this may induce more shoulder muscle activity. In addition, the duration for tablet writing should not exceed 20 minutes to avoid increased CES activation and neck discomfort.

## Supporting information

S1 File (XLSX)

S2 File (DOCX)
